# Increased Urinary 3-Mercaptolactate Excretion and Enhanced Passive Systemic Anaphylaxis in Mice Lacking Mercaptopyruvate Sulfurtransferase, a Model of Mercaptolactate-Cysteine Disulfiduria

**DOI:** 10.3390/ijms21030818

**Published:** 2020-01-27

**Authors:** Noriyuki Akahoshi, Tatsuro Minakawa, Masashi Miyashita, Uran Sugiyama, Chihiro Saito, Rintaro Takemoto, Akihiro Honda, Waka Kamichatani, Shotaro Kamata, Yasumi Anan, Isao Ishii

**Affiliations:** Department of Health Chemistry, Showa Pharmaceutical University, Machida, Tokyo 194-8543, Japan

**Keywords:** CRISPR/Cas9, cystathionine γ-lyase, hydrogen sulfide, mercaptolactate-cysteine disulfiduria, mercaptopyruvate sulfurtransferase, passive systemic anaphylaxis, reactive sulfur species, thiosulfate sulfurtransferase

## Abstract

Mercaptopyruvate sulfurtransferase (Mpst) and its homolog thiosulfate sulfurtransferase (Tst = rhodanese) detoxify cyanide to thiocyanate. Mpst is attracting attention as one of the four endogenous hydrogen sulfide (H_2_S)/reactive sulfur species (RSS)-producing enzymes, along with cystathionine β-synthase (Cbs), cystathionine γ-lyase (Cth), and cysteinyl-tRNA synthetase 2 (Cars2). MPST deficiency was found in 1960s among rare hereditary mercaptolactate-cysteine disulfiduria patients. Mpst-knockout (KO) mice with enhanced liver Tst expression were recently generated as its model; however, the physiological roles/significances of Mpst remain largely unknown. Here we generated three independent germ lines of Mpst-KO mice by CRISPR/Cas9 technology, all of which maintained normal hepatic Tst expression/activity. Mpst/Cth-double knockout (DKO) mice were generated via crossbreeding with our previously generated Cth-KO mice. Mpst-KO mice were born at the expected frequency and developed normally like Cth-KO mice, but displayed increased urinary 3-mercaptolactate excretion and enhanced passive systemic anaphylactic responses when compared to wild-type or Cth-KO mice. Mpst/Cth-DKO mice were also born at the expected frequency and developed normally, but excreted slightly more 3-mercaptolactate in urine compared to Mpst-KO or Cth-KO mice. Our Mpst-KO, Cth-KO, and Mpst/Cth-DKO mice, unlike semi-lethal Cbs-KO mice and lethal Cars2-KO mice, are useful tools for analyzing the unknown physiological roles of endogenous H_2_S/RSS production.

## 1. Introduction

Mercaptolactate-cysteine disulfiduria (MCDU; OMIN 249650) is an autosomal recessive metabolic disorder that was first identified in the late 1960s [1]. A 47-year-old man, the product of a sibling mating, was severely mentally retarded and tended to have increased susceptibility to infection. He also excreted high concentrations of 3-mercaptolactate-cysteine disulfide ((2*R*)-2-amino-3-[(2-carboxy-2-hydroxyethyl) disulfanyl] propanoic acid; C(C(C=O)O)N)SSCC(C(=O)O)O) in his urine [2,3]. The dietary supplementation of cysteine (not methionine) to this patient caused a considerable increase in the urinary excretion of the disulfide in his urine [1,4], suggesting that his disorder was associated with cysteine metabolism. Later, mentally retarded [5] and mentally normal [6] MCDU patients were identified. At the time, MCDU was considered attributable to the deficiency of 3-mercaptopyruvate sulfurtransferase (Mpst) [5]. However, the incidence rate of MCDU is not known because it is not among the lists of the newborn screening or other routine clinical tests and experimental evidence for the onset of MCDU by Mpst deficiency has yet to be demonstrated. The first Mpst-knockout (KO) mice were generated by Nagahara et al. [7]. Unfortunately, their mice displayed unexpected overexpression of the evolutionarily- [8] and catalytically-related [9] enzyme thiosulfate sulfurtransferase (Tst; idiomatically known as rhodanese/rhodanase) which has Mpst activity [10], and their urine samples were not biochemically analyzed.

Mpst mediates the metabolism of 3-mercaptopyruvate (the deamination product of cysteine) and sulfite to pyruvate and thiosulfate [11], as well as the detoxification of cyanide (CN^–^) to thiocyanate (SCN^–^) [12,13]. Mpst is also a known anti-oxidant enzyme which regulates thioredoxin peroxidase activity [8,14,15]. However, the physiological significance of Mpst remains to be elucidated. As one of the four endogenous hydrogen sulfide (H_2_S)/reactive sulfur species (RSS)-producing enzymes (along with cystathionine β-synthase [Cbs], cystathionine γ-lyase [Cth], and cysteinyl-tRNA synthetase 2 [Cars2]) [14,16,17,18,19,20,21,22], Mpst studies are now attracting attention as to their contribution to endogenous H_2_S/RSS production.

In the present study, we examined Mpst-KO mice produced using CRISPR/Cas9 technology. We preserved normal TST expression by minimizing the size of Mpst gene deletion and found that our mice display increased urinary excretion of 3-mercaptolactate and enhanced passive systemic anaphylaxis (PSA), while maintaining hepatic Tst expression/activity. Moreover, we also generated Mpst/Cth-double knockout (DKO) mice as a useful tool to investigate their mutually compensatory roles in mice.

## 2. Results

### 2.1. Establishment of Three Independent Mpst Mutant Lines

**Figure 1:**
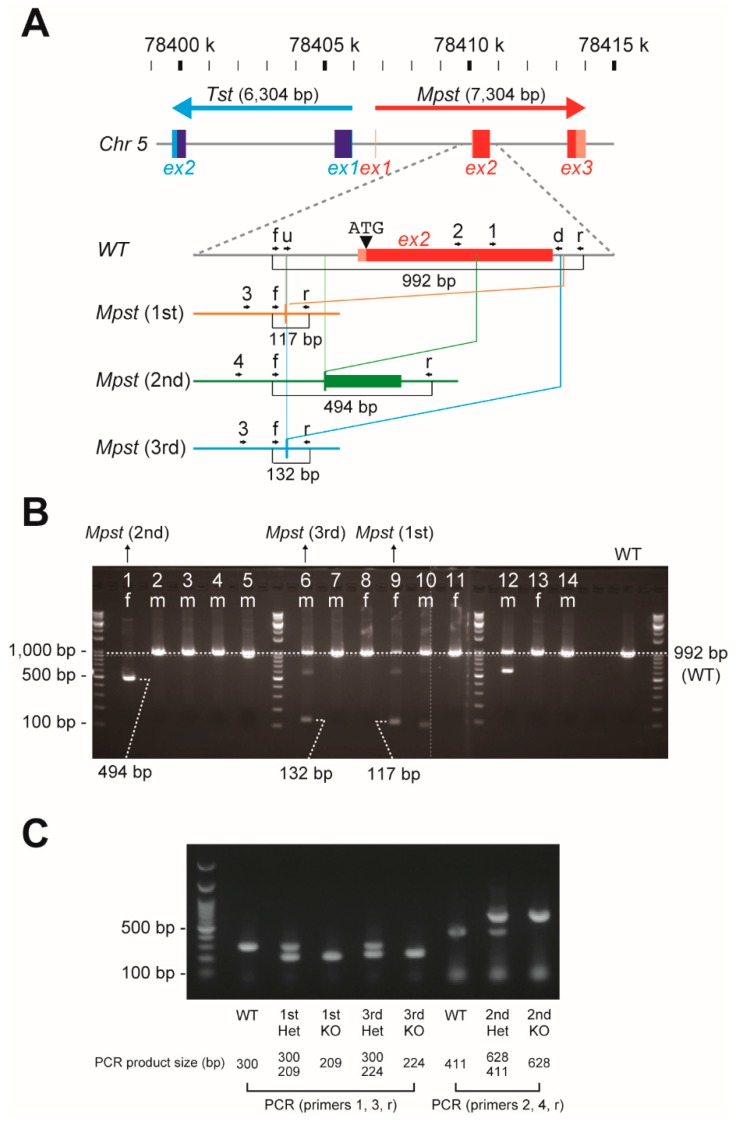
*Mpst* gene targeting in mice. (**A**) Outline for the *Mpst* gene deletion by CRISPR/Cas9 and production of 3 mutants. The 7304 bp mouse *Mpst* gene consists of 3 exons and is located proximal to its homolog *Tst* gene. The upstream (u) and downstream (d) crRNAs were designed to delete exon 2 which contains the start ATG codon and 67% of the entire open reading frame. Three independent mouse lines (1st, 2nd, and 3rd) were established. (**B**) Initial screening of 1st–3rd mouse lines from 14 independent mice (9 males and 5 females) that originated from individual fertilized zygotes electroporated with Cas9 protein, tracrRNA and crRNAs (u and d). PCR with forward (f) and reverse (r) primers detected the deletion of *Mpst* exon 2 in the 1st–3rd lines. (**C**) PCR detection of 1st and 3rd-type deletion using 1, 3, and r primers and 2nd-type deletion using 2, 4, and r primers from tail DNAs of wild-type (WT), Mpst-heterozygous (Het), and Mpst-homozygous (KO) mutant mice.

The CRISPR/Cas9 genome editing was used to delete exon 2 of *Mpst* which encodes 67% of the *Mpst* open reading frame (Figure 1A; and Supplementary Figure S1A for full DNA sequences) in one hundred C57BL/6J fertilized zygotes; from which 14 mice (9 males and 5 females) were born (14% birthrate). The Mpst deletion was apparent in two females and single male as revealed by tail DNA PCR (Figure 1B) and confirmed by direct sequencing. The targeted region was deleted in the 1st and 3rd lines but a substantial portion of random DNA repair was found in the 2nd line (Supplementary Figure S1B–E). All three lines were successful in germline transmission. Mating of their progeny produced both heterozygous and homozygous KO mice (Het and KO, respectively) as manifested by tail DNA PCR (Figure 1C). Mpst-Het and Mpst-KO mice were generally obtained with the expected frequency without marked sexual bias (Table 1).

### 2.2. Mpst, Tst, Cbs, and Cth Expression in Mpst Mutant Livers

**Figure 2:**
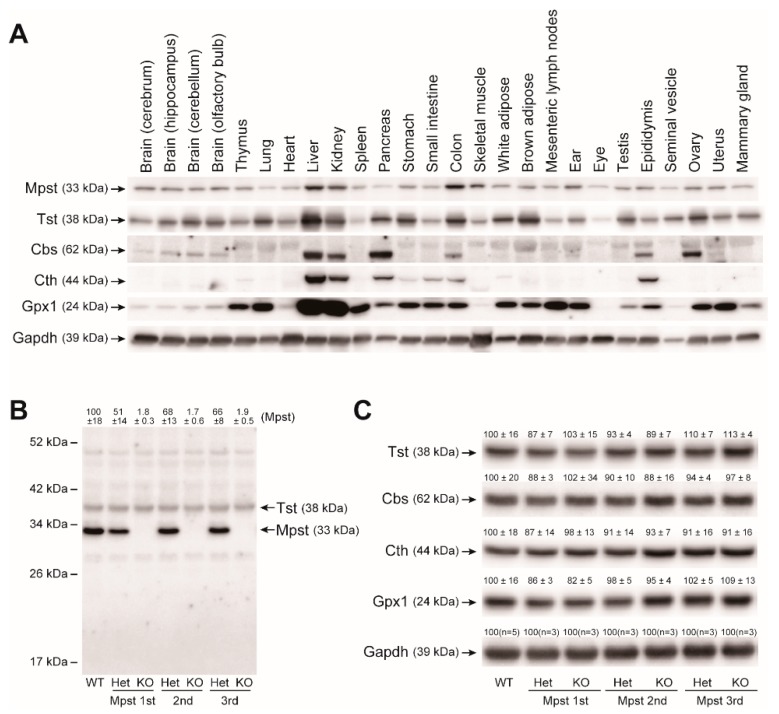
Tissue expression of mercaptopyruvate sulfurtransferase (Mpst) and related proteins in adult wild-type and Mpst mutant mice. All samples were taken from males except for the ovary, uterus, and mammary gland. (**A**) Western blot analysis of Mpst, Tst (thiosulfate sulfurtransferase), Cbs (cystathionine β-synthase), Cth (cystathionine γ-lyase), Gpx1 (glutathione peroxidase 1), and Gapdh (glyceraldehyde-3-phosphate dehydrogenase; as a loading control) in various mouse tissues. (**B**) Western blot analysis of the liver Mpst (and Tst) in wild-type (WT), heterozygous (Het), and homozygous (KO) Mpst mutant mice (1st–3rd lines) using the anti-mouse Mpst rabbit polyclonal antibody produced in this study. Relative amounts of Mpst protein was expressed as % of the WT samples (mean ± SD; *n* = 3 each). (**C**) Hepatic expression of Tst, Cbs, Cth, Gpx1, and Gapdh using specific antibodies in WT, Het, and KO mice (1st–3rd lines). Relative expression of each protein was expressed as % of the WT samples (mean ± SD; *n* = 3 each).

**Figure 3:**
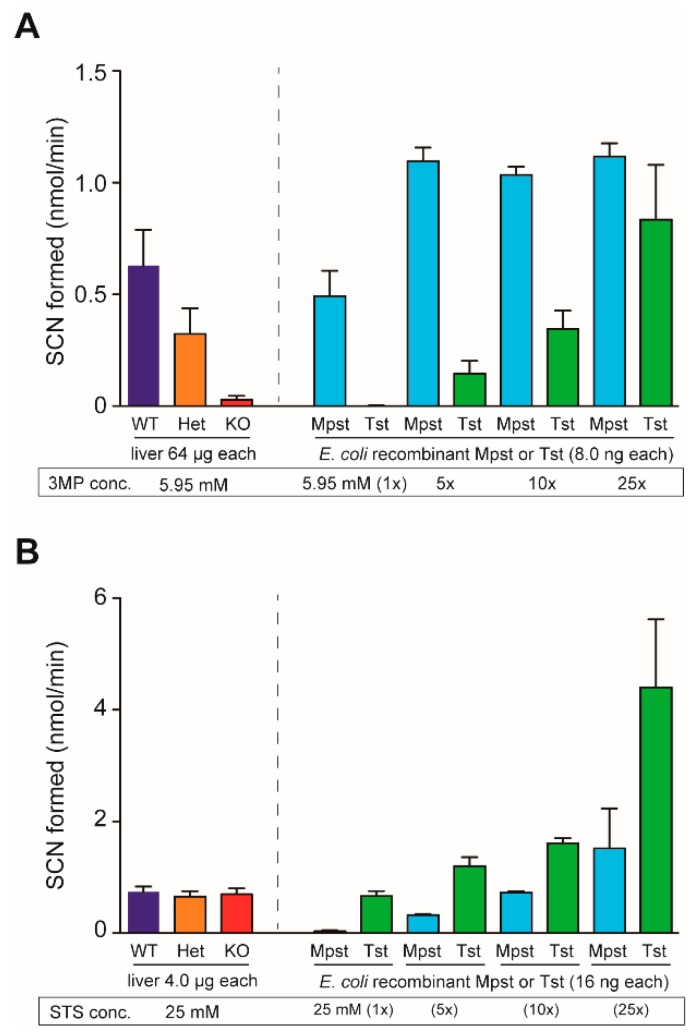
Mpst and Tst (rhodanese) enzyme activities from wild-type (WT), heterozygous (Het), and homozygous (KO) Mpst mutant mice liver homogenates, as well as mouse Mpst/Tst recombinant proteins. (**A**) Mpst enzyme assay. Although recombinant Tst protein displayed some 3-mercaptopyruvate (3-MP) degradation “Mpst” activities at substrate concentrations over 29.75 mM (5 ×), it did not show any activity at 5.95 mM (1 ×). Under this condition, *Mpst* gene deletion abolished Mpst-specific activities in liver homogenates from KO mice. (**B**) Tst enzyme assay. Although recombinant Mpst protein displayed some sodium thiosulfate (STS) degradation “Tst” activities at >125 mM (5 ×), it did not show any activity at 25 mM (1 ×). At this condition, Mpst gene deletion did not alter Tst-specific activities at any STS concentrations tested in liver homogenates from KO mice.

We developed an anti-mouse Mpst rabbit polyclonal antibody by immunizing a rabbit with purified mouse full-length recombinant Mpst protein used in our assays. Mpst expression was widespread similar to its homolog Tst. All 26 mouse organs we tested expressed 33-kDa Mpst proteins (as well as 38-kDa Tst) to greater or lesser degrees. This is in stark contrast to the other two H_2_S/RSS-producing enzymes, Cbs and Cth (Figure 2A). In all 3 germ lines, Mpst-Het livers displayed approximately 50% Mpst expression levels of wild-type (WT) livers. Mpst-KO livers did not express Mpst, as expected (Figure 2B). Therefore, the random DNA repair in the 2nd line (Supplementary Figure S1D) likely did not confer the expression of unexpected Mpst fragments. We found our Mpst antibody cross-reacts with 38-kDa Tst because pre-incubation of this antibody with recombinant Tst proteins abolished 38-kDa bands but not 33-kDa Mpst bands in the western analyses (Supplementary Figure S2). Using this antibody, we observed that hepatic Tst expression levels are not altered in all Mpst mutants (Figure 2B). Furthermore, Western blot analyses of liver proteins using a specific anti-Tst rabbit monoclonal (that does not cross-react with mouse Mpst), anti-Cbs rabbit polyclonal, anti-Cth mouse monoclonal, and anti-glutathione peroxidase 1 (Gpx1, another major anti-oxidant protein) antibodies, did not detect any significant differences for Tst, Cbs, Cth, Gpx1 expression within Mpst mutant mice (Figure 2C). Mpst and Tst are homologous proteins (63.5% similarity cDNA, 57.6% similarity protein sequence). Further, Mpst has some Tst activity and Tst has some Mpst activity [9]. Indeed, in vitro Mpst and Tst enzyme assays using 3-mercaptopyruvate (3-MP) and thiosulfate as substrates revealed that Mpst recombinant protein has some Tst activity while Tst recombinant protein has some Mpst activity at high substrate concentrations (Figure 3A,B). However, at lower concentrations (5.95 mM 3-MP for Mpst and 25 mM thiosulfate for Tst), both enzymes displayed specific activities (Figure 3A,B). At these reaction conditions and substrate concentrations, liver homogenates from Mpst mutant mice displayed specific Mpst and Tst activities that match well with their Mpst and Tst protein expression levels (Figure 3A,B).

### 2.3. Increased Urinary Excretion of 3-Mercaptolactate in Mpst-KO Mice

Serum amino acid/thiol compound levels for all lines of Mpst-KO mice were indistinguishable from those of WT mice, which was in marked contrast to Cth mice; however, all Mpst-KO mice excreted 5.5–7.3 times the normal amount of 3-mercaptolactate (3-ML) in urine (Table 2). Though not significant, urinary (total) cysteine excretion tended to be higher in all Mpst-KO mice when comparted to WT mice (Table 2). These results suggest that Mpst-KO mice displayed MCDU. In contrast, serum biochemistry (albumin, alanine aminotransferase (ALT), aspartate aminotransferase (AST), blood urea nitrogen (BUN), creatine phosphokinase (CPK), creatinine (CRE), lactate dehydrogenase (LDH), total bilirubin (T-bilirubin), total protein (T-protein), uric acid (UA), and Thiobarbituric Acid Reactive Substances activity (TBARS)) did not reveal significant differences between WT and our Mpst-KO mice lines (Table 3), suggesting generally normal organ function, including the liver and kidney.

### 2.4. Generation of Mice Lacking Both Mpst and Cth

**Figure 4:**
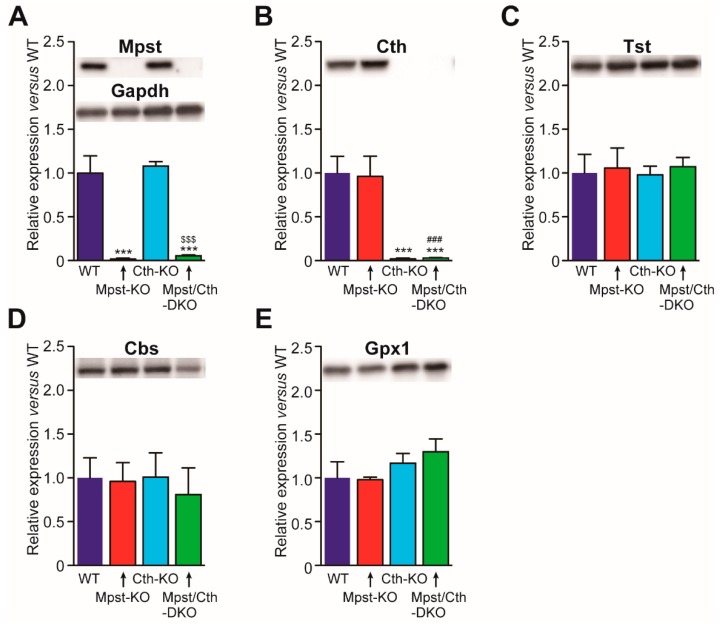
Hepatic expression of Mpst, Cth, Tst, Cbs, and Gpx1 in mice lacking Mpst, Cth, or both. Expression of Mpst (**A**), Cth (**B**), Tst (**C**), Cbs (**D**), and Gpx1 (**E**) in the livers of wild-type WT, Mpst (1st)-KO, Cth-KO, and Mpst (1st)/Cth-DKO mice was compared using Gapdh (A) as a loading control with that in WT liver as 1. Significant differences were observed versus WT mice at *** *p* < 0.001; Mpst (1st)-KO mice at ^###^ *p* < 0.001; and Cth-KO mice at ^$$$^ *p* < 0.001 (*n* = 3).

We have previously generated Cth-KO mice [23] that require cyst(e)ine as an essential amino acid [23]; display increased vulnerability to cadmium/methyl mercury [24], paraquat [23], acetaminophen [25], dietary methionine [26], cardiac ischemia/reperfusion injury [27], unilateral ureteral obstruction-induced kidney fibrosis [28], and decreased contraction responses to oxytocin [29]. Physiologically, they appear normal under laboratory conditions and are fertile [23]. Therefore, we crossbred Mpst (1st)-KO mice with Cth-KO mice to generate Mpst/Cth-DKO mice. Mpst/Cth-DKO mice were obtained at the expected frequencies without sexual bias (Table 1) and developed normally with some slightly altered serum biochemical parameters for (hepatic) functions (e.g., albumin, ALT, AST, CPK, LDH, and T-protein) when compared to the Mpst (1st)-KO mice (Table 3). We observed that Cth gene deletion only affects Cth expression while Mpst gene deletion only affects Mpst expression in the liver when compared to Tst, Cbs, and Gpx1 (Figure 4). This indicates that compensatory expression of these proteins by Mpst/Cth deletion did not occur; although slight increases in hepatic TBARS activity were observed in Mpst/Cth-DKO mice (Table 3). Even breeding between Mpst/Cth-DKO males and females, as well as Mpst-het/Cth-KO males and females, could produce substantial numbers of pups although nearly halves of the breeding resulted in the deaths of all neonates, which is attributable to phenotypes (defective lactation) in Cth-KO mothers [29].

### 2.5. Enhanced PSA Response in Mpst-KO Mice

**Figure 5:**
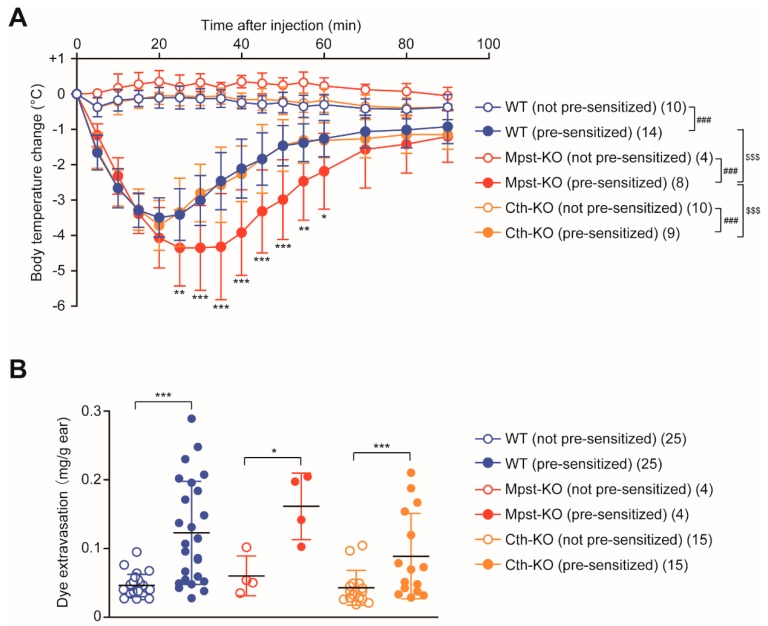
Hepatic Altered passive anaphylaxis (PSA but not PCA) in Mpst-KO mice. (**A**) Analysis of rectal temperatures in IgE-antigen-dependent PSA for WT, Mpst (1st)-KO, and Cth-KO mice after antigen challenge. Differences between pre-sensitized versus not pre-sensitized (vehicle alone) measurements were significant at ^###^ *p* < 0.001 as well as those between genotypes at ^$$$^ *p* < 0.001 in a two-way repeated-measured ANOVA with Bonferroni’s multiple comparison test (Differences in each time point were significant at * *p* < 0.05, ** *p* < 0.01, and *** *p* < 0.001). (**B**) Analysis of ear edema in IgE-antigen-induced PCA in WT, Mpst-KO, and Cth-KO mice after antigen challenge. Differences in Evans blue dye extravasation between left (not pre-sensitized) and right ears (pre-sensitized with anti-DNP IgE) were evaluated. The ratio differences were significant (* *p* < 0.05 and *** *p* < 0.001, pre-sensitized versus not pre-sensitized) in ratio paired *t*-tests. Differences in the ratios were not significant between the genotypes in a one-way ANOVA with Tukey’s multiple comparison test.

H_2_S has been shown to regulate various inflammatory responses [30,31], but its implications on anaphylaxis, “a serious generalized or systemic, allergic or hypersensitivity reaction that can be life-threatening or fatal [32,33],” remain unknown. Here we investigated dinitrophenyl (DNP)-conjugated human serum albumin (HSA)-induced PSA and passive cutaneous anaphylaxis (PCA) after pre-sensitization with anti-DNP IgE in WT, Mpst-KO, and Cth-KO mice as described in Materials and Methods. First, PSA was induced and changes in rectal temperature were followed. The hypothermic response associated with PSA was more pronounced in Mpst-KO mice compared to WT or Cth-KO mice; the maximum response was greater and the recovery was delayed (Figure 5A). Next, PCA was induced and differences in Evans blue dye extravasation between left (not pre-sensitized) and right ears (pre-sensitized with anti-DNP IgE) were evaluated. In contrast to PSA, PCA responses were similar in all mice (Figure 5B).

## 3. Discussion

MCDU has historically been attributed to MPST deficiency, but studies containing direct experimental evidence are lacking. We provide here the first direct evidence that Mpst deficiency in mice causes enhanced secretion of 3-ML in urine, a hallmark trait of MCDU (Table 2). Cysteine is first metabolized to 3-MP by AST. Following, 3-MP is metabolized to pyruvate by Mpst or to 3-ML by LDH; and thus, Mpst deletion could lead to enhanced 3-ML excretion to urine [11]. The quantitative detection of 3-mercaptolactate-cysteine disulfide in urine samples is currently underway. MCDU patients are rarely found; thus, previous investigations into 3-ML or 3-mercaptolactate-cysteine disulfide quantities in MCDU patient urine samples have yet to be performed. Therefore, we do not know whether the 5.5–7.3-fold level increase of 3-ML in urine (Table 2) is mirrored in MCDU patients.

Previously generated Mpst-KO mice exhibited increased anxiety-like behaviors associated with increased levels of serotonin and its major metabolite (5-hydroxyindoleacetic acid) in the prefrontal cortex and decreased levels of dopamine in the hippocampus [7]. Further, 2–3-fold increases in hepatic (mitochondrial) Tst expression have been observed [10]. Because Tst has some Mpst activity (that can metabolize 3-ML, Figure 3 and [9]), the potential overexpression of Tst may have an unexpected influence (e.g., alterations in neurotransmitter levels) on behavioral phenotypes in Mpst-KO mice. In this regard, our Mpst-KO mice lines have a great advantage over previous KO mice as hepatic Tst expression levels in our mice were comparable to that of WT mice (Figure 2B,C). Indeed, in our preliminary analyses, our Mpst-KO mice were free of any behavioral disorders common to all mutant lines such as increased anxiety (data not shown), which is in good agreement with a previous report that showed the absence of obvious physical and mental disabilities in two MCDU sisters (11 and 13 years old) [6]. Expression of Cbs, Cth, and Gpx1 were also comparable between all Mpst-KO mice and WT mice (Figure 2C). It should be noted that hepatic expression of Gpx1 (the anti-oxidant “selenoprotein”) was not altered. This is important because Mpst may participate in selenium delivery to selenoproteins [34]. Accordingly, hepatic GSSG/tGSH ratios as well as TBARS activities (as the measures of oxidative stress/injury) in Mpst-KO mice were equivalent to those of WT mice (Table 2). The phenotypic differences between previous Mpst-KO mice and our Mpst-KO mice could be solely attributable to different *Mpst* gene deletion strategies as the mouse Tst gene is located near the Mpst gene (Figure 1A) and their genetic backgrounds are identical (C57BL/6J) [7].

In this study, we analyzed, for the first time, the systemic anaphylactic responses in Cth-KO and Mpst-KO mice and found greater PSA (but not PCA) responses in Mpst-KO mice (Figure 5). In anaphylaxis, mast cells and macrophages secrete various inflammatory mediators including histamine, platelet-activating factor, and cysteinyl leukotrienes; thereby eliciting vasodilation and increased vascular permeability which leads to hypothermia [35,36]. Passive anaphylactic responses originate from mast cells that are pre-sensitized with antigen-specific IgE antibodies [33]. Because vascular permeability levels (in PCA assay) were equivalent between Mpst-KO and WT mice, enhanced hypothermic responses (in PSA assay) are likely caused by abnormal vasodilation and autonomous thermoregulation rather than mast cell responses. A recent study demonstrated that the administration of propargylglycine into mice, a non-specific inhibitor of Cth, interferes with LPS (lipopolysaccharide)-induced and brown adipose tissue-mediated thermogenesis [37]; suggesting a possible role for H_2_S/RSS in general thermoregulation. Further studies are necessary to delineate the roles of Mpst/Cth and produced H_2_S/RSS in the thermoregulation.

Furthermore, we generated Mpst/Cth-DKO mice that develop normally (with generally normal serum biochemical parameters; Table 3) which are fertile. Although Cth is located upstream of Mpst in cysteine metabolism, both enzymes could provide active sulfane sulfur from cysteine in parallel for cyanide detoxification to thiocyanate [13]. General incidences for inherited diseases of amino acid metabolism by single gene deletion are estimated to be less than 1/250,000 [38]; thus, the occurrence of MCDU combined with “Cth-deficient” cystathioninuria should be extremely rare. Nonetheless, Mpst/Cth-DKO mice as well as Mpst-KO and Cth-KO mice are a very useful in vivo analytical tool, which contrasts with (semi-)lethal Cbs-KO [39,40] and Cars2-KO mice [22]. Mpst/Cth-DKO mice generally displayed the combined phenotypes of Mpst-KO (increased 3-ML levels in urine; Table 2) and Cth-KO mice (e.g., increased serum levels of His, cystathionine, citrulline, and total homocysteine (Hcy); increased urinary levels of total Cys, total Hcy, total cysteinylglycine (Cys-Gly), and total γ-glutamylcysteine (γGlu-Cys); and reduced total glutathione (GSH) and increased GSSG/total GSH ratios in the liver) (Table 2). We previously reported that Cth-KO mice look normal but display severe disorders upon various intervention, and our Mpst-KO mice and Mpst/Cth-DKO mice may be the similar. Although we are experiencing technical difficulties in obtaining sufficient numbers of Cth-KO pups from Cth-KO mothers due to defective milk ejection [29] irrespective of coexisting *Mpst* mutations, further studies including the examination of Mpst/Cth-DKO mice for PSA/PCA alterations are needed.

In conclusion, we generated Mpst-KO mice as an animal model of MCDU and also Mpst/Cth-DKO mice as a useful tool for analyzing functions of H_2_S/RSS that both enzymes may produce in vivo. Moreover, we found a novel phenotype in Mpst-KO mice, the enhanced passive systemic anaphylaxis responses. Physiological significance and roles of Mpst are yet unknown and the further studies using those mice (e.g., molecular mechanisms of enhanced PSA in Mpst-KO mice) may provide new insight into the sulfur amino acid metabolism.

## 4. Materials and Methods 

### 4.1. Generation of Mpst (1st–3rd)-KO Mice

Mpst-KO mice were generated using CRISPR/Cas9 technology from Setsurotech Inc. (Tokushima, Japan) on a C57BL/6J (C57BL/6JJcl; CLEA Japan, Tokyo, Japan) background as described previously [41] with some modifications. Briefly, in vitro fertilized zygotes (C57BL/6J × C57BL/6J) were electroporated with 100 ng/µL recombinant Cas9 protein, 100 ng/µL tracrRNA, and 100 ng/µL of two crRNAs targeted upstream and downstream of the *Mpst* gene: *Mpst* crRNA upstream (u) (5′-ACGACCGCGGGCGCCAGGAG-3′) and *Mpst* crRNA downstream (d) (5′-GCTGGGGAAACGGACTAATG-3′). Electroporated zygotes were transferred into the oviducts of pseudopregnant female ICR mice (Jcl:ICR, CLEA Japan), and a total of 14 mice were born on E19.5. Correct targeting/deletion was confirmed by PCR using forwarding (f) (5′-TGCTGGGCAAGGAATAGAGTG-3′) and reverse (r) (5′-GCATATAAGGCCAGCACACG-3′) primers that are located outside the targeted locus (Figure 1B). We sequenced the amplified PCR products for 1st, 2nd, and 3rd lines to confirm the deletion (Supplementary Figure S1). Routine tail DNA genotyping was done by three primer PCR reactions using primer 1 (5′-TTCCGCCACTGGCTGAATCAG-3′), primer 3 (5′-AGCTGGGCTACATCTTAGAGA-3′), and the reverse primer for the 1st and 3rd lines; and primer 2 (5′-ACGCACGTCGTGATCTACGAC G-3′), primer 4 (5′-CCTAGACCGGCTCAATGAATGT -3′), and the reverse primer for the 2nd line (Figure 1C). Adult (8–18 weeks of age) male mice were used for our analyses. Sperms and embryos from all Mpst-KO mice lines were deposited in the Center for Animal Resources and Development (CARD) at Kumamoto University (Kumamoto, Japan; http://card.medic.kumamoto-u.ac.jp/card/english/index.html). All animal procedures conformed to the Guide for the Care and Use of Laboratory Animals, 8th Edition published by the US National Research Council and were approved by the Animal Care Committees of Showa Pharmaceutical University (No. P-2016-10 and P-2018-07; approval dates: 22 July 2016 and 12 April 2018, respectively).

### 4.2. Preparation of Mpst and Tst Recombinant Proteins

His-tagged murine Mpst (CS-Mm29353-B01-01; GenBank (NM_001162492.1)) and Tst (CS-Mm05875-B01-01; GenBank (NM_009437.4)) expression plasmids were purchased from GeneCopoeia (Rockville, MD, USA). The plasmids were transformed to Rosetta (DE3) pLysS Competent Cells (Novagen (Merck)). Single colonies were picked up from LB agar plates containing 100 µg/mL Ampicillin (Amp) and 34 µg/mL Chloramphenicol (CP), and placed into 75 mL LB media (100 µg/mL Amp and 34 µg/mL CP) for growth overnight at 37 °C. 50-mL of the overnight culture was seeded into 1-L LB media (100 µg/mL Amp) and grown at 30 °C for 1.5 h and at 20 °C for 2 h. Following, isopropyl β-D-galactopyranoside (IPTG) was added (final 0.5 mM) and the culture was further grown at 20 °C for 24 h. Cells were then centrifuged and resuspended in 40 mL buffer E (20 mM Tris-HCl (pH 8.0), 150 mM NaCl, 1 mM Tris(2-carboxyethyl)phosphine (TCEP) hydrochloride, and 10% (v/v) glycerol) containing a Complete EDTA-free Protease Inhibitor Cocktail (Roche Diagnostics, Tokyo, Japan). Cells were then lysed by sonication and the soluble fraction was isolated by centrifugation (12,000× *g*, 4 °C, 20 min). Mpst and Tst recombinant proteins were affinity-purified with the TALON Metal Affinity Resins (Clontech) and a linear gradient of 10–100 mM imidazole. The pooled fraction was dialyzed twice against buffer E at 4 °C overnight using a Slide-A-Lyzer G2 Dialysis Cassette–20kDa cutoff (Thermo Fisher Scientific). The recombinant proteins were stored at −80 °C until use.

### 4.3. Rabbit Polyclonal Mpst Antibody Production

A single Japanese white rabbit (3.0 kg female; Slc:JW/CSK (Japan SLC, Shizuoka, Japan)) was immunized with full-length mouse Mpst recombinant proteins using Freund’s complete adjuvant four times (day 0 (0.3 mg), 28 (0.2 mg), 35 (0.2 mg), and 42 (0.2 mg)) and whole blood was collected at day 49. The blood was allowed to clot by leaving undisturbed at room temperature for 20 min, and the clot was removed by centrifugation at 2000× *g* for 10 min at 4 °C. A total of 54 mL resulting supernatant (i.e., antiserum/polyclonal antibody) was obtained. Efficient antibody titers (O.D. > 3.0 at 1:16,000 dilution) were confirmed by ELISA (data not shown).

### 4.4. Western Blot Analyses

Each mouse tissue sample (~100 mg) was homogenized in lysis buffer (1 mL *per* 100 mg; 50 mM Tris-HCl (pH 7.4), 150 mM NaCl, 1.0 mM EDTA, 1% (w/v) Triton X-100, 0.1% (w/v) phenylmethylsulfonyl fluoride, and Complete EDTA-free Protease Inhibitor Cocktail) using a MicroSmash-100R homogenizing system (Tomy, Tokyo, Japan) and zirconia beads (4100 rpm, 30 s × 8, 4 °C). Homogenates were centrifuged at 15,300× *g*, at 4 °C for 20 min. Supernatants were recentrifuged at 15,300× *g*, at 4 °C for 30 min. After heating (95 °C, 5 min) with the sample buffer, samples (5 µg/lane) were resolved on 10% or 12% pre-cast PAGE gels, and then transferred to the Immobilon PVDF membrane (Millipore) and subjected to western blotting. Mpst (and Tst as a cross-reactive protein) were detected with the above polyclonal antibody (1:10,000 in Can Get Signal immunostain Solution 1 (Toyobo)) and anti-rabbit IgG, HRP-linked whole Ab donkey antibody (GE Healthcare; 1:10,000 in Tris-Buffered Saline-Tween 20). Tst was detected with anti-human TST rabbit monoclonal antibody (Abcam ab1666625; 1:10,000) and anti-rabbit IgG, HRP-linked whole Ab donkey antibody (GE Healthcare; 1:10,000). Cbs was detected with anti-rat CBS rabbit polyclonal antibody ([42]; 1:5000) and anti-rabbit IgG, HRP-linked whole Ab donkey antibody (1:10,000). Cth was detected with anti-human CTH mouse monoclonal antibody (Abnova H00001491-M01; 1:5000) and mouse IgGκ BP-HRP (mouse IgGκ light chain binding protein-horseradish peroxidase; Santa Cruz sc-516102; 1:10,000). Gpx1 was detected with anti-human Gpx1/2 mouse monoclonal antibody (SantaCruz sc-133160; 1:10,000) and mouse IgGκ BP-HRP (1:10,000). Gapdh was detected as a loading control with anti-GAPDH rabbit monoclonal antibody (Cell Signaling Technology (Danvers, MA, USA) #2118; 1:10,000) and anti-rabbit IgG, HRP-linked whole Ab donkey antibody (GE Healthcare; 1:10,000). Chemiluminescence detection was performed using Chemi-Lumi One Ultra (Nacalai Tesque, Kyoto, Japan) and the ATTO WSE-6100 LuminoGraph I imager (ATTO, Tokyo, Japan).

### 4.5. Mpst and Tst Activity Assays

Mpst enzyme assays were performed using the substrate 3-MP as described previously [43,44], with some modifications. Liver homogenates (64 µg each) or recombinant proteins (8.0 ng each) were incubated on 96-well microplates with 5.95 (1×)–148.75 mM (25×) 3-MP, 175.6 mM 2-amino-2-methylpropane-1,3-diol (AMPD), and 16.1 mM potassium cyanide (KCN) in an 84 µL mixture at 25 °C for 30 min. The reaction was stopped by adding 20 µL of 38% formaldehyde (final 6.67%) and processed by adding 96 µL of Goldstein’s ferric nitrate reagent (Fe(NO_3_)_3_ + HNO_3_ + H_2_O; [45]). Thiocyanate (SCN^–^) formation was determined by the measurement of absorbance at 460 nm using the Epoch 2 microplate spectrophotometer (BioTek/Agilent, Winooski, VT, USA). Tst enzyme assays were performed using the substrate thiosulfate as described previously [45] with some modifications. Liver homogenates (4.0 µg each) or recombinant proteins (16 ng each) were incubated with 25 (1 ×)–625 mM (25 ×) thiosulfate, 47.6 mM KH_2_PO_4_, and 71.4 mM KCN at 25 °C for 30 min and processed similarly. Specific Mpst and Tst activities were obtained at substrate concentrations of 5.95 mM (for 3MP) and 25 mM (for thiosulfate), respectively.

### 4.6. Measurements of Amino Acid/Thiol Compound Levels and Biochemical Parameters

Serum samples were analyzed for their amino acid concentrations using amino acid and thiol-derivatization fluorescent reagents (4-fluoro-7-nitrobenzofurazan (NBD-F) and 4-fluoro-7-sulfobenzofurazan (SBD-F), respectively; Dojindo, Kumamoto, Japan) via a high-performance liquid chromatography (HPLC) as previously described [46] (Supplementary Figures S3–S5). Urine samples were analyzed for 3-ML and other thiol compounds via SBD-F and HPLC using a modified column/mobile phase system (to separate 3-ML and Cys-Gly peaks) as described previously [47,48] (Supplementary Figure S6). Briefly, SBD-F-labeled samples (2 µL) were injected onto the InertSustain Amide column (3.0 mm ID × 150 mm; GL Science, Tokyo, Japan), initialized at 10% A (50 mM ammonium formate (pH 3.0))/90% B (acetonitrile) for 30 min, followed by 25% A/75% B for 15 min, and finalized by 10% A/90% B for 15 min, using an Agilent 1100 series chromatography workstation (Agilent, Santa Clara, CA, USA). The peaks in fluorescence (Ex. 385 nm; Em. 515 nm) were measured using an Agilent 1260 fluorescence detector. All amino acids/thiol compounds were identified based on their retention times; concentrations were calculated relative to the calibrated standard solutions. Serum biochemical parameters (albumin, ALT, AST, BUN, CPK, CRE, LDH, T-bilirubin, T-protein, and UA) were measured using a SpotChem EZ SP-4430 clinical analyzer (Arkray, Tokyo, Japan). Hepatic levels of oxidized glutathione (GSSG) and total glutathione (GSH+GSSG) were measured using the GSSG/GSH Quantification Kit (Dojindo, Kumamoto, Japan). GSSG/total glutathione percentages were calculated as a measure of oxidative status. TBARS assays were performed using TBARS assay kit from Cayman Chemicals (Ann Arbor, MI, USA) on serum and liver homogenates as per the manufacturer’s instructions.

### 4.7. PSA and PCA Assays

PSA and PCA assays were performed as previously described [49]. For PSA assays, WT, Mpst-KO, and Cth-KO mice were intravenously administered with 3 µg of anti-2,4-dinitrophenyl (DNP) mouse monoclonal IgE (SPE-7; Sigma-Aldrich) in 200 µL PBS or PBS alone through tail veins. At 24 h, the mice were challenged intravenously with 500 µg of DNP-conjugated human serum albumin (DNP-HSA (filtered through 0.22-μm filter (Millipore) before use); Sigma-Aldrich) in 200 µL PBS. After allergen challenge, rectal temperature was monitored every 5 min for 2 h using a BAT-12R microprobe thermometer equipped with a Mouse RET ISO Rectal Probe (Physitemp Instruments, Clifton, NJ, USA). For our PCA assays, mice were passively sensitized by intradermal injection with 10 ng of anti-DNP mouse monoclonal IgE (SPE-7) in 20 µL PBS. After 24 h, the mice were challenged by intravenous injection (through tail veins) with 20 µg of DNP-HSA in 200 µL saline containing 1 mg of Evans blue dye. Evans blue dye extravasation in the ear after 30 min was evaluated after sacrifice. Ears were removed and incubated at 37 °C overnight in 500 µL of 1 N KOH. The lysates were then mixed with 17.05 µL of 44 N phosphoric acid and 975 µL of acetone, and quantitative analysis of the dye in the extracts was performed within a linear range from 0.2 to 25 µg measuring the absorbance at 620 nm.

### 4.8. Statistical Analyses 

Data were expressed as mean ± SD (n: sample numbers). Statistical comparison was performed using Prizm 5 software (GraphPad, San Diego, USA). Two-way repeated-measured ANOVA with Bonferroni’s multiple comparison test was used in PSA assays (Figure 5A) and ratio (pre-sensitized/not pre-sensitized) paired *t*-test was used in PCA assays (Figure 5B). One-way ANOVA with Tukey’s multiple comparison tests were used in all other experiments. All *P* values less than 0.05 denoted a significant difference.

## Figures and Tables

**Table 1:** Inheritance of the *Mpst* and *Cth* mutant alleles in mice.

Parental Genotypes							Genotypes of Offsprings (≥3-week-old)											
Male		×	Female		Total Litter No.	Average Litter Size	Mpst-WT				Mpst-Het				Mpst-KO			Sex Ratio Male:Female
*Mpst*	*Cth*		*Mpst*	*Cth*			Cth-WT	Cth-Het	Cth-KO		Cth-WT	Cth-Het	Cth-KO		Cth-WT	Cth-Het	Cth-KO	
**Mpst single mutant**																		
Het (1st)			WT		27	5.1 ± 2.6	59				78							52:85
Het (1st)			Het (1st)		81	5.7 ± 2.2	114				234				112			244:216
KO (1st)			KO (1st)		5	5.2 ± 1.8									26			16:10
Het (2nd)			WT		14	5.5 ± 2.0	37				40							41:36
Het (2nd)			Het (2nd)		17	5.0 ± 2.3	28				36				21			35:50
KO (2nd)			KO (2nd)		10	4.0 ± 2.0									40			21:19
Het (3rd)			WT		9	3.6 ± 2.4	16				16							16:16
Het (3rd)			Het (3rd)		30	5.1 ± 2.0	30				73				49			83:69
KO (3rd)			KO (3rd)		15	5.4 ± 2.4									81			39:42
**Mpst (1st)/Cth double mutant**																		
Het	KO		Het	KO	11	4.6 ± 1.6			12				25				14	27:24
KO	Het		KO	Het	29	5.8 ± 2.4									50	87	32	88:81
KO	KO		KO	KO	8	3.6 ± 1.9											29	18:11

Total litter numbers, average litter size (mean ± SD), and numbers of genotyped offsprings from the indicated crosses are shown.

**Table 2:** Concentrations of serum amino acid/thiol compounds, urinary thiol compounds, and liver glutathione in adult wild-type, Mpst (1st–3rd)-KO, Cth-KO, and Mpst (1st)/Cth-DKO mice.

		Mpst				Mpst (1st)/Cth
Mice	WT (a)	1st KO (b)	2nd KO	3rd KO	Cth-KO (c)	-DKO (d)
Serum (µM)	(*n* = 10)	(*n* = 7)	(*n* = 7)	(*n* = 7)	(*n* = 5)	(*n* = 10)
Ala	479 ± 128	377 ± 56	439 ± 79	430 ± 88	574 ± 239	407 ± 88
Arg	120 ± 19 ^c^	104 ± 23 ^c^	120 ± 14	113 ± 24	168 ± 37 ^abd^	146 ± 26 ^c^
Asn/Asp	6.28 ± 2.66 ^c^	9.33 ± 6.68 ^c^	17.4 ± 9.4	6.01 ± 4.41	28.1 ± 17.8 ^abd^	14.3 ± 6.4 ^c^
Gln	807 ± 135	752 ± 133	762 ± 74	801 ± 35	935 ± 172 ^d^	654 ± 147 ^c^
Glu	27.4 ± 21.4 ^c^	26.1 ± 17.9 ^c^	36.7 ± 15.7	19.9 ± 8.1	54.5 ± 17.9 ^ab^	39.2 ± 10.7
Gly	328 ± 75	291 ± 38	352 ± 28	306 ± 64	368 ± 109	345 ± 49
His	42.0 ± 24.5 ^cd^	44.2 ± 23.3 ^cd^	85.1 ± 19.1	39.3 ± 20	131 ± 58 ^ab^	90.4 ± 10.4 ^ab^
Ile	109 ± 18	106 ± 18	113 ± 21	105 ± 14	123 ± 20	106 ± 20
Leu	159 ± 21	155 ± 22	170 ± 26	169 ± 15	196 ± 11 ^d^	156 ± 32 ^c^
Lys	217 ± 37	226 ± 42	211 ± 48	255 ± 33	298 ± 24	184 ± 44
Met	75.3 ± 19.4	69.9 ± 16.7 ^c^	86.9 ± 8.0	67.6 ± 11.0	104 ± 25 ^b^	96.6 ± 22
Phe	95.6 ± 26.9	79.5 ± 13.9	86.0 ± 7.0	89.8 ± 6.2	106 ± 23	83.3 ± 13.4
Pro	131 ± 68	91.2± 26.7	120 ± 30	94.5 ± 28.4	177 ± 89	135 ± 36
Ser	145 ± 58	120 ± 18	143 ± 18	125 ± 29	175 ± 62	141 ± 30
Thr	175 ± 42	151 ± 35	180 ± 21	163 ± 45	199 ± 35	194 ± 43
Trp	71.2 ± 17.1	73.9 ± 11.1	61.5 ± 16.5	62.9 ± 12.0	69.9 ± 9.6	82.9 ± 22.3
Tyr	94.8 ± 32.0	87.6 ± 25.0	110 ± 18	101 ± 26	99.2 ± 58.9	80.3 ± 30.4
Val	242 ± 58	225 ± 40 ^c^	253 ± 29	226 ± 27	304 ± 43 ^b^	251 ± 41
Cystathionine	44.1 ± 19.7 ^cd^	39.5 ± 12.8 ^cd^	56.2 ± 12.6	37.6 ± 8.1	124 ± 15 ^ab^	133 ± 32 ^ab^
Citrulline	79.2 ± 19.3 ^cd^	63.0 ± 15.2 ^cd^	68.6 ± 14.1	67.2 ± 12.7	169 ± 24 ^ab^	135 ± 34 ^ab^
Ornithine	42.1 ± 10.9 ^c^	35.1 ± 10.2 ^c^	35.7 ± 6.6	43.0 ± 16.2	66.4 ± 19.1 ^ab^	49.5 ± 13.8
Taurine	675 ± 349	491 ± 136	642 ± 181	514 ± 88	601 ± 240	664 ± 235
Total Cys	319 ± 40 ^d^	322 ± 52 ^d^	341 ± 78	315 ± 32	274 ± 11	261 ± 51 ^ab^
Total Hcy	5.78 ± 1.22 ^cd^	5.90 ± 2.05 ^cd^	5.58 ± 0.77	6.23 ± 1.11	83.6 ± 18.6 ^abd^	121 ± 15 ^abc^
Total GSH	252 ± 71 ^cd^	230 ± 141 ^d^	221 ± 42	224 ± 37	124 ± 59 ^a^	107 ± 42 ^ab^
Total Cys-Gly	3.23 ± 0.39 ^b^	4.43 ± 0.59 ^ac^	4.79 ± 0.55	3.93 ± 0.39	3.15 ± 0.93 ^b^	3.87 ± 0.86
Total γ Glu-Cys	10.4 ± 2.8	13.2 ± 4.0 ^cd^	12.1 ± 2.4	8.37 ± 1.94	8.17 ± 0.45 ^b^	8.32 ± 2.16 ^b^
Urine (mmol/mol creatinine)						
	(*n* = 10)	(*n* = 9)	(*n* = 7)	(*n* = 7)	(*n* = 6)	(*n* = 9)
3-ML	10.4 ± 2.0 ^bd^	61.3 ± 9.9 ^acd^	57.4 ± 29.4	75.9 ± 20.7	16.9 ± 3.2 ^bd^	91.2 ± 21.0 ^abc^
Total Cys	156 ± 28 ^cd^	187 ± 24 ^cd^	222 ± 50	211 ± 84	624 ± 272 ^ab^	601 ± 115 ^ab^
Total Hcy	7.32 ± 2.66 ^cd^	6.24 ± 2.81 ^cd^	6.15 ± 1.11	5.44 ± 1.24	169 ± 51 ^ab^	195 ± 69 ^ab^
Total GSH	2.00 ± 1.28	1.40 ± 0.81	2.31 ± 1.09	1.74 ± 0.44	2.67 ± 1.84	2.15 ± 1.09
Total Cys-Gly	26.6 ± 10.0 ^cd^	25.4 ± 7.1 ^cd^	41.3 ± 9.6	32.4 ± 12.0	85.8 ± 37.7 ^ab^	82.4 ± 33.9 ^ab^
Total γ Glu-Cys	10.2 ± 4.9 ^cd^	9.45 ± 3.70 ^cd^	21.9 ± 7.0	14.3 ± 3.5	24.3 ± 13.0 ^ab^	26.8 ± 7.7 ^ab^
Liver						
Total GSH (nmol/mg protein; *n* = 5 each)						
	10.6 ± 1.5 ^cd^	10.9 ± 1.7 ^cd^	N.T.	N.T.	6.51 ± 0.75 ^ab^	6.25 ± 1.25 ^ab^
GSSG/tGSH (%; *n* = 5 each)						
	4.04 ± 0.48 ^cd^	3.82 ± 0.48 ^cd^	N.T.	N.T.	5.42 ± 0.62 ^ab^	5.69 ± 0.89 ^ab^

Means ± SD are presented (*n*: sample numbers). N.T., not tested. All parameters were similar in Mpst-KO mice lines; therefore, statistical analyses were done between WT, Mpst (1st)-KO, Cth-KO, and Mpst (1st)/Cth-DKO mice by one-way ANOVA with Tukey’s multiple comparison test. Significant differences (*p* < 0.05) versus WT (a), Mpst (1st)-KO (b), Cth-KO (c), and Mpst (1st)/Cth-DKO mice (d).

**Table 3:** Biochemical parameters in serum (and liver only for TBARS activity) for adult wild-type, Mpst (1st–3rd)-KO, Cth-KO, and Mpst (1st)/Cth-DKO mice.

		Mpst				Mpst (1st)/Cth
Mice	WT (a)	1st KO (b)	2nd KO	3rd KO	Cth-KO (c)	-DKO (d)
Serum	(8)	(7)	(7)	(7)	(5)	(10)
Albumin (g/dL)	2.10 ± 0.12	2.21 ± 0.18 ^d^	2.14 ± 0.10	2.19 ± 0.20	2.00 ± 0.19	1.97 ± 0.16 ^b^
ALT (IU/L)	17.5 ± 10.8	12.1 ± 2.2 ^d^	16.3 ± 5.0	16.0 ± 5.3	22.0 ± 8.1	29.2 ± 12.6 ^b^
AST (IU/L)	45.3 ± 9.7	39.6 ± 12.3 ^d^	42.6 ± 16.6	42.1 ± 14.0	51.0 ± 20.4	75.2 ± 40.4 ^b^
BUN (mg/dL)	25.2 ± 3.3	25.4 ± 3.4	25.8 ± 1.9	26.8 ± 5.3	26.5 ± 2.5	26.5 ± 3.1
CPK (IU/L)	394 ± 110	306 ± 96 ^d^	448 ± 222	271 ± 62	351 ± 84	470 ± 154 ^b^
CRE (mg/dL)	< 0.2	< 0.2	< 0.2	< 0.2	< 0.2	< 0.2
LDH (IU/L)	173 ± 48 ^d^	157 ± 16 ^d^	282 ± 93	184 ± 28	225 ± 54	308 ± 80 ^ab^
T-bilirubin (mg/dL)	0.29 ± 0.11	0.30 ± 0.12	0.37 ± 0.11	0.27 ± 0.08	0.36 ± 0.11	0.35 ± 0.07
T-protein (g/dL)	4.59 ± 0.22 ^d^	4.71 ± 0.44 ^d^	4.39 ± 0.23	4.63 ± 0.49	4.26 ± 0.27	4.13 ± 0.23 ^ab^
UA (mg/dL)	2.81 ± 0.79 ^cd^	1.97 ± 1.12	1.74 ± 0.60	2.17 ± 0.79	1.60 ± 0.39 ^a^	1.63 ± 0.40 ^a^
	(6)	(5)			(6)	(8)
TBARS (nmol/mL)	1.68 ± 0.22	1.68 ± 0.23	N.T.	N.T.	1.87 ± 0.41	1.50 ± 0.17
Liver						
TBARS	(6)	(5)			(6)	(8)
(nmol/µg protein)	522 ± 29 ^d^	546 ± 77	N.T.	N.T.	659 ± 78	702 ± 169 ^a^

Means ± SD are presented (*n*: sample numbers). N.T., not tested. All parameters were similar in Mpst-KO mice lines; therefore, statistical analyses were done between WT, Mpst (1st)-KO, Cth-KO, and Mpst (1st)/Cth-DKO mice by one-way ANOVA with Tukey’s multiple comparison test. Significant differences (*p* < 0.05) versus WT (**a**), Mpst (1st)-KO (**b**), Cth-KO (**c**), and Mpst (1st)/Cth-DKO mice (**d**).

## Supplementary Materials

Supplementary Data can be found at https://www.mdpi.com/1422-0067/21/3/818/s1. Figure S1: Mouse Mpst gene and CRISPR/Cas9-mediated deletion of exon 2, Figure S2: Pre-incubation of anti-mouse Mpst rabbit polyclonal antibody with recombinant mouse Tst proteins abolishes 38-kDa (Tst) but not 33-kDa (Mpst) bands, Figure S3: Representative chromatograms in the measurements of free amino acids in serum samples, Figure S4: Representative chromatograms in the measurements of tryptophan in serum samples, Figure S5: Representative chromatograms in the measurements of thiol compounds in serum samples, Figure S6: Representative chromatograms in the measurements of 3-mercaptolactate in urine samples.

## Abbreviations

ALT	Alanine aminotransferase
AST	Aspartate aminotransferase
BUN	Blood urea nitrogen
Cars2	Cysteinyl-tRNA synthetase 2
Cbs	Cystathionine β-synthase
CPK	Creatine phosphokinase
CRE	Creatinine
Cth	Cystathionine γ-lyas
DKO	Double knockout
DNP	2,4-dinitrophenyl
Gpx1	Glutathione peroxidase 1
GSH	Glutathione
GSSG	Glutathione (oxidized form)
Hcy	Homocysteine
H_2_S	Hydrogen sulfide
LDH	Lactate dehydrogenase
MCDU	Mercaptolactate-cysteine disulfiduria
3-ML	3-Mercaptolactate
3-MP	3-Mercaptopyruvate
Mpst	Mercaptopyruvate sulfurtransferase
PCA	Passive cutaneous anaphylaxis
PSA	Passive systemic anaphylaxis
RSS	Reactive sulfur species
TBARS	Thiobarbituric acid reactive substances
Tst	Thiosulfate sulfurtransferase
UA	Uric acid
WT	Wild-type
