# Supplementary material for: Increased Urinary 3-Mercaptolactate Excretion and Enhanced Passive Systemic Anaphylaxis in Mice Lacking Mercaptopyruvate Sulfurtransferase, a Model of Mercaptolactate-Cysteine Disulfiduria
Source: Int J Mol Sci. 2020 Jan 27;21(3):818. doi: 10.3390/ijms21030818 (PMC7038117; doi:10.3390/ijms21030818)
Supplement: Supplementary file 1 [file ijms-21-00818-s001.pdf]

Supplementary Data

Contents

Supplementary Materials and Methods

Supplementary Figure 1. Mouse *Mpst* gene and CRISPR/Cas9-mediated deletion of exon 2.

Supplementary Figure 2. Pre-incubation of anti-mouse Mpst rabbit polyclonal antibody with recombinant mouse Tst proteins abolishes 38-kDa (Tst) but not 33-kDa (Mpst) bands.

Supplementary Figure 3. Representative chromatograms in the measurements of free amino acids in serum samples.

Supplementary Figure 4. Representative chromatograms in the measurements of tryptophan in serum samples.

Supplementary Figure 5. Representative chromatograms in the measurements of thiol compounds in serum samples.

Supplementary Figure 6. Representative chromatograms in the measurements of 3-mercaptolactate in urine samples.

Supplementary Materials and Methods

Measurements of free amino acids in serum

The amino acids in serum (as well as homophenylalanine as the internal standard and homoarginine for the injection check) were derivatized using NBD-F in accordance with the manufacturer's instructions. The samples (10 µl) were injected onto a reversed-phase C18 column (CAPCELL PAK C_{18} MGII S5; 5.0 µm, 3.0 × 150 mm; Shiseido, Tokyo, Japan) for the separation of fluorescent-derivatized amino acids. The mobile phase consisted of elution solution A (10 mM citrate buffer [pH 6.2] containing 75 mM $NaClO_4$) and B (50% [v/v] acetonitrile). A gradient of increasing solution B as follows was used: 5–10% for 20 min, 10–37% for 35 min, 37–38% for 5 min, 38–79.3% for 10 min, 79.3–90% for 5 min, and 100% for 5 min. All gradient steps were controlled by a LaChrom Elite chromatography workstation (Hitachi, Tokyo, Japan), and peaks in fluorescence (Ex. 480 nm; Em. 530 nm) were measured using an L-2400 fluorescence detector (Hitachi). All amino acids were identified based on their retention times and concentrations were calculated relative to the calibrated standard solutions (Wako, Osaka, Japan).

Measurements of free thiol compounds in serum

Total cysteine/homocysteine (Hcy)/glutathione (GSH)/cysteinylglycine (CG)/γ-glutamylcysteine (γGlu-Cys), which gives rise to their respective thiol compounds after reductive cleavage of disulfide bonds [8], were measured by the following procedure. The serum samples or standard reagents (20 µl) were mixed with 10 µl of phosphate-buffered saline (pH 7.4), 10 µl of 80 µM *N*-(2-mercaptopropionyl)glycine (MPG; internal standard), and 4 µl of 0.349 M (100 g/l) Tris(2-carboxyethyl)phosphine hydrochloride, and then incubated at room temperature for 30 min. Then the mixtures were added to 36 µL of 0.612 M (100 g/l) trichloroacetic acid (TCA)/1 mM EDTA (pH 8.0) solution and centrifuged at 13,000 *g* at 20°C for 10 min. The 50 µl supernatant was mixed with 185 µl of a mixture of 50 µg SBD-F in 175 µl of 125 mM borate-HCl (pH 9.5)/4.0 mM EDTA (pH 8.0) and 10 µl of 1.55 M NaOH, and incubated at 60°C for 1 h. The samples (10 µl) were injected onto the same C18 column using an Agilent 1100 series chromatography workstation (Agilent, Santa Clara, CA, USA) for the separation of fluorescent-derivatized thiol-containing amino acids. The mobile phase consisted of elution solution A (0.1 M sodium acetate [pH 4.5]) and B (50% [v/v] methanol). A gradient of increasing solution B as follows was used: 6% for 5 min and 6–100% for 10 min. The peaks in fluorescence (Ex. 385 nm; Em. 515 nm) were measured using an Agilent 1260 fluorescence detector. All thiol compounds were identified based on their retention times and concentrations were calculated relative to the calibrated standard solutions (Wako).

Measurements of tryptophan in serum

Tryptophan was independently quantified by its own fluorescence without NBD-F derivatization. The samples (10 µl) were injected onto the same C18 column using an Agilent 1100 series chromatography workstation. The mobile phase consisted of elution solution A (0.1 M sodium acetate [pH 4.5]) and B (50% [v/v] methanol). A gradient of increasing solution B as follows was used: 5–7.2% for 13 min. The peaks in fluorescence (Ex. 295 nm; Em. 340 nm) were measured using an Agilent 1260 fluorescence detector.

Supplementary Figure 1. Mouse *Mpst* gene and CRISPR/Cas9-mediated deletion of exon 2.

A. Mouse *Mpst* gene (total 7,304 bp)

```
1                                                                                                100
gtgcccaagtctcctccctttggctggccgcaggttggtggcggaaggagggaacctctgagggcgtggggagggggtgacatggcggagctgggaaacg
gggagcgcaaatccctggtatctgctacccaacgcgtcctacttgcttttctgtgagagagggtgcttctccggggggtgcatagcgtggtatcacgcggg
agtggaggaggggatgcagtctatgagagaagagtttccagggcgcctctaatttgccttaggaagactgtccgtggctgcttgtggccgtctgtgggtt
ggtgtgggataagagacaggtgggtagcagtgagtggatggggtttggaaagacctgatgcctcctgagaaagttgggggggggagcaagagaataaac
aaagcaattcagaggagctgtggtgaggagatctagcatgccccaagaggagaaagtaacagaacggaataaaaataaaactagcatttatgaagcccgc
cttgccgagtgccttcacgaccctgccggattgagctgaactcccaacaggagaaacaggtagagccaggaggtgccaaggcacgtagtggaaaagggac
taatttctcacctgaagcccaaagtggtgggagctgcctcggtttacctgtccatccttgaagccttatgcttagtagtagtagtagtcgtcgttgtcgt
cttgggcttcagttttaccacttcattgtaaatttatataaatcccaataagcaagatatctggaaatagagcatggctcctgcaaaccagctgtacac
cccctctctagtctattctgttcccctggaatgcttttctcttgaactcccctccctgtttttttgagcatctatcctaccctgaaatactgtcacagtg
tgttgcaaaaattcactgtctagcattttcccagcatactctgcacctggagcttgacagtagggaagagaaggtaccaggagggtctagttagggagat
gctcttcagtgcccgaaccccacccccccatgggtgggatatataggctcacactaacttaggatggcctgggcctgtcctatatgtcctgatggacttc
cagagagctactctcccctgggatcctggcccttcaagcacaaagaaacctagtctctttctggtctcttcccctcatgagcactcatagcctagcctct
ctgactctctttctgctgcctggggtcttttgtcataactgtcaatcaagcagggagggtagtgacctcagacctttgaagtcccacccccctggaagtct
aaggccagctgtagactcaagggccattaggagagtaaaggaaggaatagcaggtttgcattcaacttccctaaatgatcattcaaagaagctgtttata
gggtcagtggtggcccttctatagtcacgtttttattctcactaagaggtgggttttggagtctggcatggtggtatatgcttgtaatccacccacccccc
actcccccactcccccactctggactctgagtaggaagaggagaatttcaagttccaggatagagagtgagggaaagtgctgggatgatagctcacgtga
ttaagcgcttgccttgacagcacaaggccctgagctggatccccagagtcttcataaacaacacaaggcatgttgcatgcctgtgatccagccctgggga
aggagagacaggtagatccctaggctcagtgaccagccagccaaggtgagtcctaggccatgggaagtcccccatcccaaaaatgatataaagttaataa
aatggctggtgcctggggccccatctgagatctgagattgttctctgccccaccccaccaccttcacacaagcgcgcgtgcgcacgcacacacacgcaca
cacacacacacacacacacacacaggttggaagttccaatatacgacacgtgactcaggaaccctcacataccaacatgaatcgcccataccatgtcatc
tctgctctctgactcccaaaccagagctgaggaggcaaaattcccaagaatgatgtcagtaaaggggctactgcttcctgaaaggattgtcccacctcag
tctccttgctctctgtgcacacacccgccctcctgtgtcccaactctcacatccagctgcatcacaaacactagtaaagagtttcactctgtagcccaag
ccatcctggaactctctctgtagaccaggctagcctcacactcacagaattcgagtgctgggacaaatggtgtgtaccaccagtgcccgcccggcttagc
ccttatgcttatccctaggtataaagtgtgtctgcttcccacggaaggccttctgccttctctctccaatccaggctaaggtagaggccttgctgtgcac
actaaactccagaattcctcacttgcaccattgcttgagctgctctggggcctaccaaagtcaagagttctcaatgtgtgccccccaccgccaccccacac
agggcctaggccagtgatgcaaacctatccccacttagtttctgttgtctatttgtggggttttgttgttattgttgttctagtttttgtttgtttgatt
gattggttttgtttattttgttttgagctggtcttgctgagtagcccgggctggtctggaacttcctatgtagactaggctggccctgacctctggtcct
tctacctcaggctcttgagtattataggaggtacatctactaactcaggtaattcaaagcatatcagggagcctcggtggctttgagaaaaagatgacag
tacctaacctcttaagggtgttccctcagcaggctggggagaggctgctgagtcaggtgttgtaataaatgaggattaaatgtttctgggcaaagggtga
attctggttatgttcccattggactcctagaccggctcaatgaatgtgtggatctgattggctgtgaagctgggctacatcttagagaagggctgaaact
ttggtcttatttgctttacctatgggctaattgagcctggcccgggctctaccctactttgctgggcaaggaatagagtgagggacacgcaccaccctct
cctggcgcccgcggtcgtaactgttctttgaagccatgcattattcaaagcctggcaggggggctccctgggtgttaaaggcagagcctggctcacccaag
gccctgtctgagttcttggactctgctctgccggtacgcaggctccacctacaccgattagcacatatccctagtcgcaatccctgaatgcaggcttgtc
tctccattctggtatcttccctgttttgcagccccgcaatctgagcatctcctaggccatggccgccccgcaacttttccgagctctggtgtctgcgcag
tgggtggcggaggctctgaaggccccgcgctcctcacagccgctgaagttactggacgcgtcctggtacctacccaagctgggccgcgatgcacgacgcg
agtttgaggagcgccacatcccgggcgccgccttcttcgacatcgatcgttgcagtgaccacacgtcccccctacgaccacatgctgcctaacgccacgca
ctttgccgactacgcgggcagcttgggcgtgagcgccgccacgcacgtcgtgatctacgacgacagtgaccagggcctctattccgctccgcgggtctgg
tggatgttccgcgccttcggccaccactctgtgtcattgctggacggcggcttccgccactggctgaatcagaacctgccaatcagctccggcaaaagcc
actcggagcccgcggagttcagcgcgcagctcgatccctctttcatcaagacccacgaggacatcctggagaacttggatgcccggcgcttccaggtggt
ggacgcccgcgcagctggccgtttccaaggcacccagccagaaccccgagatggtaacgcgctggggaaacggactaatggggatgtggggactggccgg
tgtgtgatgtgcgctccagacactgcctagccgtgtgctggccttatatgcatacagagcaaagggataagtgttggttgctgggcgcttcactctaagc
atctcgctttcttcttatagtggaggccacacactacctcactgttttggaactctgggctgaggttgcctgagtaccttgagaatccagggcacccagg
ccaggacagcccactaactgtaatactgagcttgtgtacagccaccacaaaagagcagaaggcagcccgcacctggcttaggaaggacccaagctttgtc
ctgatgagatctagcctgcagctcctgagttgatggggtgcttgctgtacacctgcaactgccacctaggtaatattaagagattctgagagggtattag
gatgggtcagtcgctcaaaacggttcgtgccccaagtgagggcaaagctggctgcctatagcctgggtttctccaggcaaagcccagtgtaatatgggaa
aggatctcacagagcaagctgggctatctatagccatgctagtgtgagccaaatctctcaggagctaggtagagccagccttcagtatctgcagacacca
gatttcagtgtttaaacaacctcagaaaacatttcctgtacagagggtatgccctttcttgccctgtctattcctgaatgacacagtgtaactgtatagc
attcacaactgtgatggatattgaaagagatctagagatgggtgcaacactgcatggctgttatcccagcatttggaggctgagacaggtggatcaccag
tttgaggccagcctgggctacatagtgatttccaggccagcctgaactagagagtaagatcctgtctcaaaactcaaagggagagaaaggagttgggga
gactctagagatgtgttcagtgttaggagggtgcacgtagattgcatgacaggacaccatttcatgtgatttggtgtgtgtgtaggggggggggggtaca
ggcactagttctcctgaaaatatggagcaatacccacactgacacagagcaggtgaggagggtagccctggagtggctgccttttctgagatcttaggaa
ggagcctctctggattcagactccctgcatacttaccaacagtgagcaaccagtagttctccctaccacttctgtccagactgccaggtgggagctagc
catggcccccaaagctctcccatgccctctctgctggtccagaattctgttcagagccctgtctctgctgtactttgcatagcacctttggatggtgacc
cttgcatgtgtagaatgaaagcagtaggtgagagttaaagtgcttcgagccactgggctggaagccgtactagtctttacttaaagcactaccatgtctc
ctccgcagtccatgacttggcagagtacacctgtaatttgctccttcagtccagcaggtcagggtttgtgttttgcttggtcaagctggaggtataagga
agtcctgactctacttacccataggaccctggctggagagctagctcttctccaggctttgagaagggctgctcagggtgactttaggagacccacttc
actggggatggccaacacactgaatcctctcaaagctcagaagcactgtctctggccatcctctgagaatagagactgccacggaccatcagctcctgca
aagacagggaaacaggtagaggtctctcacacactcatattgggatctagatggcccagatttgtcggacagctatgaggtttcattctgatgtgactct
cagccctcctgggaaaatggagctttaataacctctggctggaagaaagctagagacaaaatacatcctctacctccctattgtctgagccttggtggct
gggatgcctgttgcatcatcccagagttcctgggttctttctgtctgccaggctctcagtcacactgtcaggactcttgattggatagtttcattctggg
agaagaccctaccctccctaggcaccgaatggcagcacggcctctggggtggttaccactcccccctacccactgctagctagagcatgctagtctttgtg
ccttcttctcctggggtgggggtgacagacatttccttcccatagaatttgggtggtattaaaggagaaaacgctgcagaggggggtctaggcagtatcct
gcacaacacatgtgttcagccatgtcagccatttgattgctatttttaaccatagccctagggtatttctgtcacactggcaagtatctggttatcgcc
tgtctgccctctatgccatctctaaccatccaagtgtcctgagtcctaattggacagaggtcatgtgtactactctgctgcccctcaagattcacggtca
ggaagcccagtgtgttgtgtagctctggtagagtctagttcttaggttcaaactctagccagcaattagctgggtacagtgactttgggtgtgacttcgc
cttaggctggccatctgtgtgtggggcggtgcctaactctgctggctggatgataagacattggacacacatttgccattctgctgtggtggaagatctg
ataccacaaataccaccaaccctagatatcactcatagtgtggccttccctgtgttcccccgtgcaggcatcgaacctggccacatccctggctcagtaa
acatcccgttcactgaattcctgaccaacgaaggcctggagaagagcccagaggagatcaaacgcctgtttaaggagaagaaggtggacctgtctaagcc
cttggtagccacgtgtggctccggtgtcacagcctgccacgtggtcctgggggccttcctctgtggcaagtcagacgtgcctgtctatgatggctcctgg
gtagagtggtacatgcgtgcccagccagagcatatcatctctgagggccgagggaagacccagtgaagccaggcaggacacagtgcagcttgggtgacat
ctgaccatcacagtaccagccactggtcactctgaccctgctttgccaagagtcctttctcagacaactgagggagtgtttggggtgatatcccagagac
ctggagttccagtgactcatagatactcagctgaggtagccaagaaggtggcaggcagtaagagggtacccaggggagggagggagggagggaggaaggg
aggaagggagggaggccagatagcaaaacagcttgcacggtgctaagctggggcccacctagcttgtttcattgttgggaaataaaacatcaagaacaaa
aaca
```

Exon, CDS, Intron, Exon1 variation, CRISPR-cleavage site

B. WT allele

```
2901                                                                                             3000
attctggttatgttcccattggactcctagaccggctcaatgaatgtgtggatctgattggctgtgaagctgggctacatcttagagaagggctgaaact
ttggtcttatttgctttacctatgggctaattgagcctggcccgggctctaccctactttgctgggcaaggaatagagtgagggacacgcaccaccctct
cctggcgcccgcggtcgtaactgttctttgaagccatgcattattcaaagcctggcaggggggctccctgggtgttaaaggcagagcctggctcacccaag
gccctgtctgagttcttggactctgctctgccggtacgcaggctccacctacaccgattagcacatatccctagtcgcaatccctgaatgcaggcttgtc
tctccattctggtatcttccctgttttgcagccccgcaatctgagcatctcctaggccatggccgccccgcaacttttccgagctctggtgtctgcgcag
tgggtggcggaggctctgaaggccccgcgctcctcacagccgctgaagttactggacgcgtcctggtacctacccaagctgggccgcgatgcacgacgcg
agtttgaggagcgccacatcccgggcgccgccttcttcgacatcgatcgttgcagtgaccacacgtcccccctacgaccacatgctgcctaacgccacgca
ctttgccgactacgcgggcagcttgggcgtgagcgccgccacgcacgtcgtgatctacgacgacagtgaccagggcctctattccgctccgcgggtctgg
tggatgttccgcgccttcggccaccactctgtgtcattgctggacggcggcttccgccactggctgaatcagaacctgccaatcagctccggcaaaagcc
actcggagcccgcggagttcagcgcgcagctcgatccctctttcatcaagacccacgaggacatcctggagaacttggatgcccggcgcttccaggtggt
ggacgcccgcgcagctggccgtttccaaggcacccagccagaaccccgagatggtaacgcgctggggaaacggactaatggggatgtggggactggccgg
tgtgtgatgtgcgctccagacactgcctagccgtgtgctggccttatatgcatacagagcaaagggataagtgttggttgctgggcgcttcactctaagc
```

C. Mpst 1st-type allele

```
2901                                                                                             3000
attctggttatgttcccattggactcctagaccggctcaatgaatgtgtggatctgattggctgtgaagctgggctacatcttagagaagggctgaaact
ttggtcttatttgctttacctatgggctaattgagcctggcccgggctctaccctactttgctgggcaaggaatagagtgagggacacgcaccaccctct
cc--------------------------------------------------------------------------------------------------
----------------------------------------------------------------------------------------------------
----------------------------------------------------------------------------------------------------
----------------------------------------------------------------------------------------------------
----------------------------------------------------------------------------------------------------
----------------------------------------------------------------------------------------------------
----------------------------------------------------------------------------------------------------
----------------------------------------------------------------------------------------------------
-----------------------------------------------------------------------------atggggatgtggggactggccgg
tgtgtgatgtgcgctccagacactgcctagccgtgtgctggccttatatgcatacagagcaaagggataagtgttggttgctgggcgcttcactctaagc
```

D. Mpst 2nd-type allele

```
2901                                                                                             3000
attctggttatgttcccattggactcctagaccggctcaatgaatgtgtggatctgattggctgtgaagctgggctacatcttagagaagggctgaaact
ttggtcttatttgctttacctatgggctaattgagcctggcccgggctctaccctactttgctgggcaaggaatagagtgagggacacgcaccaccctct
c-tggcgcccgcggtcgtaactgttctttgaagccatgcattattcaaagcctggcaggggggctccctgggtgttaaaggcagagcctggctcacccaag
gccctgtctgagttcttggactctgct-------------------------------------------------------------------------
----------------------------------------------------------------------------------------------------
----------------------------------------------------------------------------------------------------
----------------------------------------------------------------------------------------------------
----------------------------------------------------------------------------------------------------
----------------tcggccaccactctgtgtcattgctggacggcggcttccgccactggctgaatcagaacctgccaatcagctccggcaaaagcc
actcggagcccgcggagttcagcgcgcagctcgatccctctttcatcaagacccacgaggacatcctggagaacttggatgcccggcgcttccaggtggt
ggacgcccgcgcagctggccgtttccaaggcacccagccagaaccccgagatggtaacgcgctggggaaacgg--------ggatgtggggactggccgg
tgtgtgatgtgcgctccagacactgcctagccgtgtgctggccttatatgcatacagagcaaagggataagtgttggttgctgggcgcttcactctaagc
```

E. Mpst 3rd-type allele

```
2901                                                                                             3000
attctggttatgttcccattggactcctagaccggctcaatgaatgtgtggatctgattggctgtgaagctgggctacatcttagagaagggctgaaact
ttggtcttatttgctttacctatgggctaattgagcctggcccgggctctaccctactttgctgggcaaggaatagagtgagggacacgcaccaccctct
cctggAGGCTCCGGTGTC----------------------------------------------------------------------------------
----------------------------------------------------------------------------------------------------
----------------------------------------------------------------------------------------------------
----------------------------------------------------------------------------------------------------
----------------------------------------------------------------------------------------------------
----------------------------------------------------------------------------------------------------
----------------------------------------------------------------------------------------------------
----------------------------------------------------------------------------------------------------
------------------------------------------------------------------------------tggggatgtggggactggccgg
tgtgtgatgtgcgctccagacactgcctagccgtgtgctggccttatatgcatacagagcaaagggataagtgttggttgctgggcgcttcactctaagc
```

Exon, CDS, Intron, CRISPR-cleavage site

Primers

Mpst-forward (sense orientation)
Mpst-reverse (anti-sense orientation)
Mpst-1 (sense orientation)
Mpst-2 (sense orientation)
Mpst-3 (sense orientation)
Mpst-4 (sense orientation)

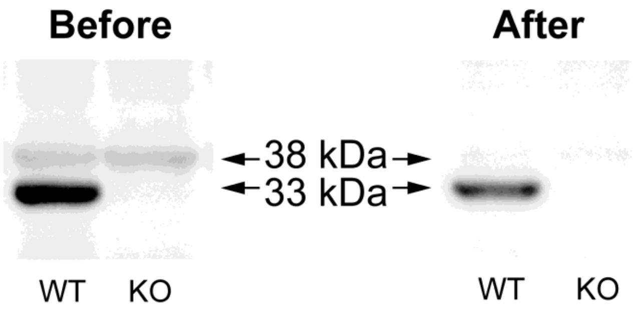

Supplementary Figure 2. Pre-incubation of anti-mouse Mpst rabbit polyclonal antibody with recombinant mouse Tst proteins abolishes 38-kDa (Tst) but not 33-kDa (Mpst) bands. His-tagged mouse Tst recombinant proteins were pre-incubated with anti-mouse Mpst antiserum in PBS at 4°C overnight, and the mixture was passed through the TALON Metal Affinity Resins to remove Tst (not Mpst)-associated antibody. Hepatic Mpst and Tst proteins in WT and Mpst(1st)-KO mice were detected with anti-mouse Mpst antibody (Before) or that pre-incubated with Tst recombinant proteins (After).

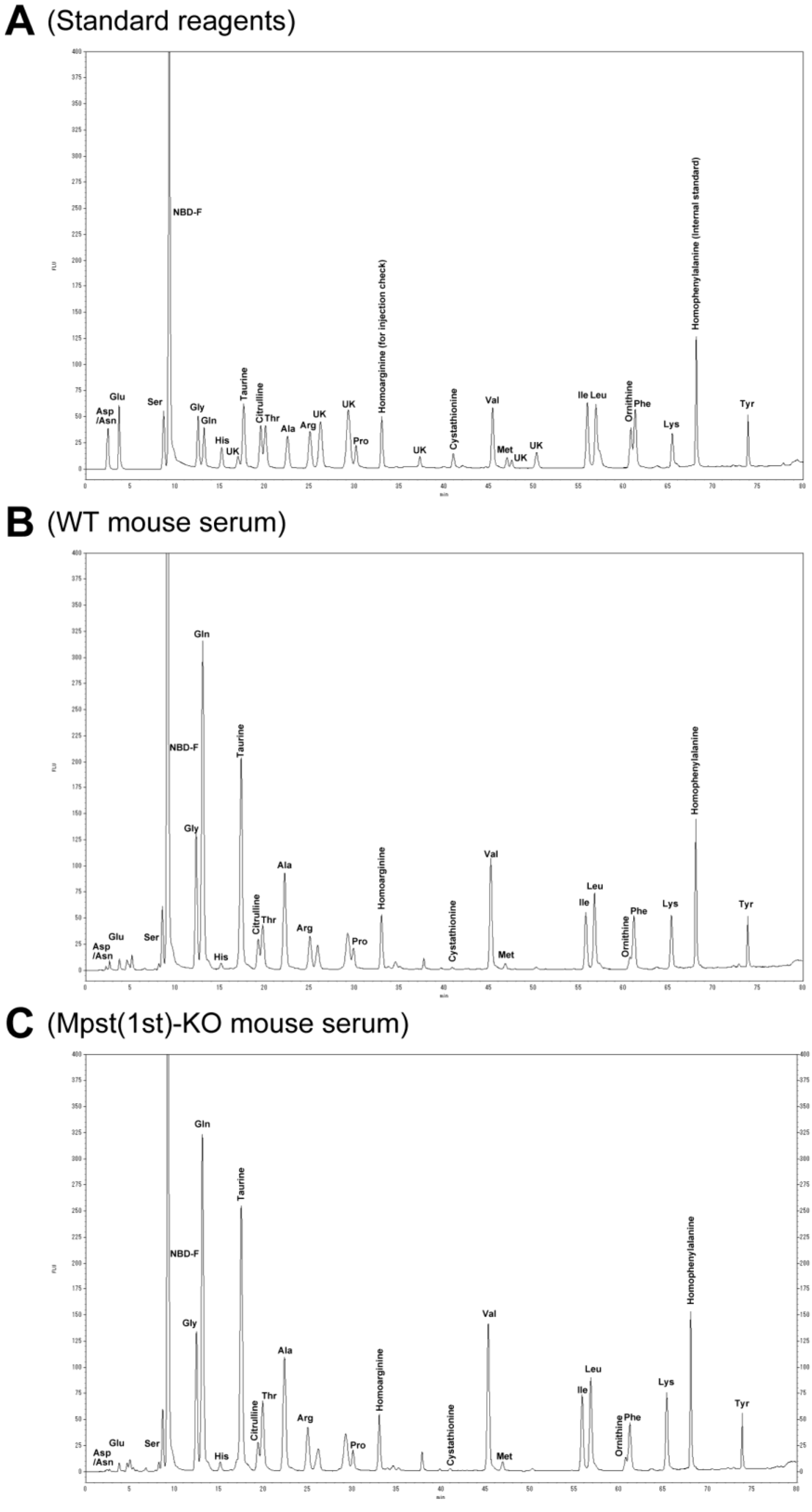

Supplementary Figure 3. Representative chromatograms in the measurements of free amino acids in serum samples. Standard amino acid reagents (A), WT mouse serum (B), and Mpst (1st)-KO mouse serum (C), were analyzed by NBD-F labeling and HPLC coupled with a fluorescence detector. UK, unknown. Homophenylalanine peaks were used as the internal standard and homoarginine peaks for the injection check. The concentrations of the standard reagents used in (A) are 250 µM for cystathionine and 125 µM for other amino acids. Most unknown peaks seem to be derived from the degradation products of unreacted NBD-F.

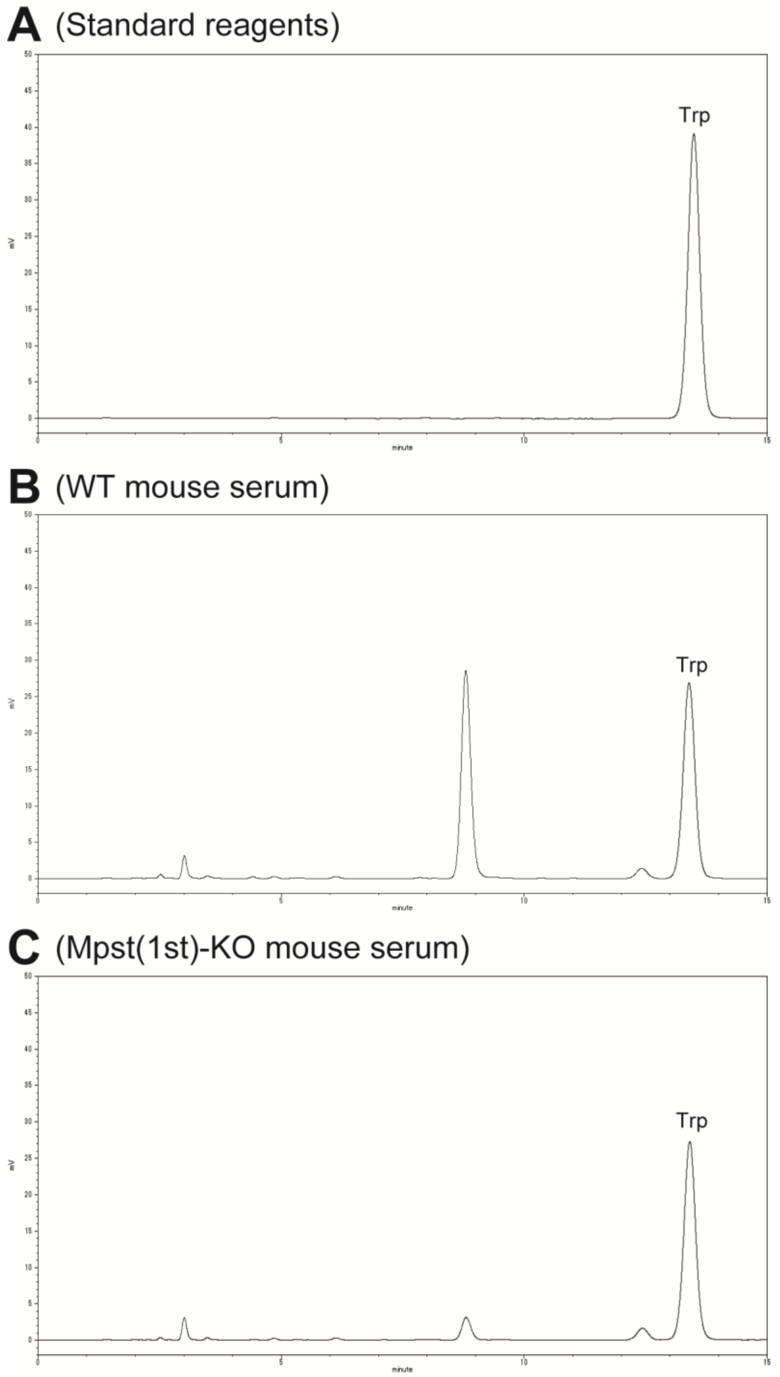

Supplementary Figure 4. Representative chromatograms in the measurements of tryptophan (Trp) in serum. Standard Trp reagent (A), WT mouse serum (B), and Mpst (1st)-KO mouse serum (C), were analyzed by HPLC coupled with a fluorescence detector. The concentration of the standard Trp reagent in (A) is 100 µM.

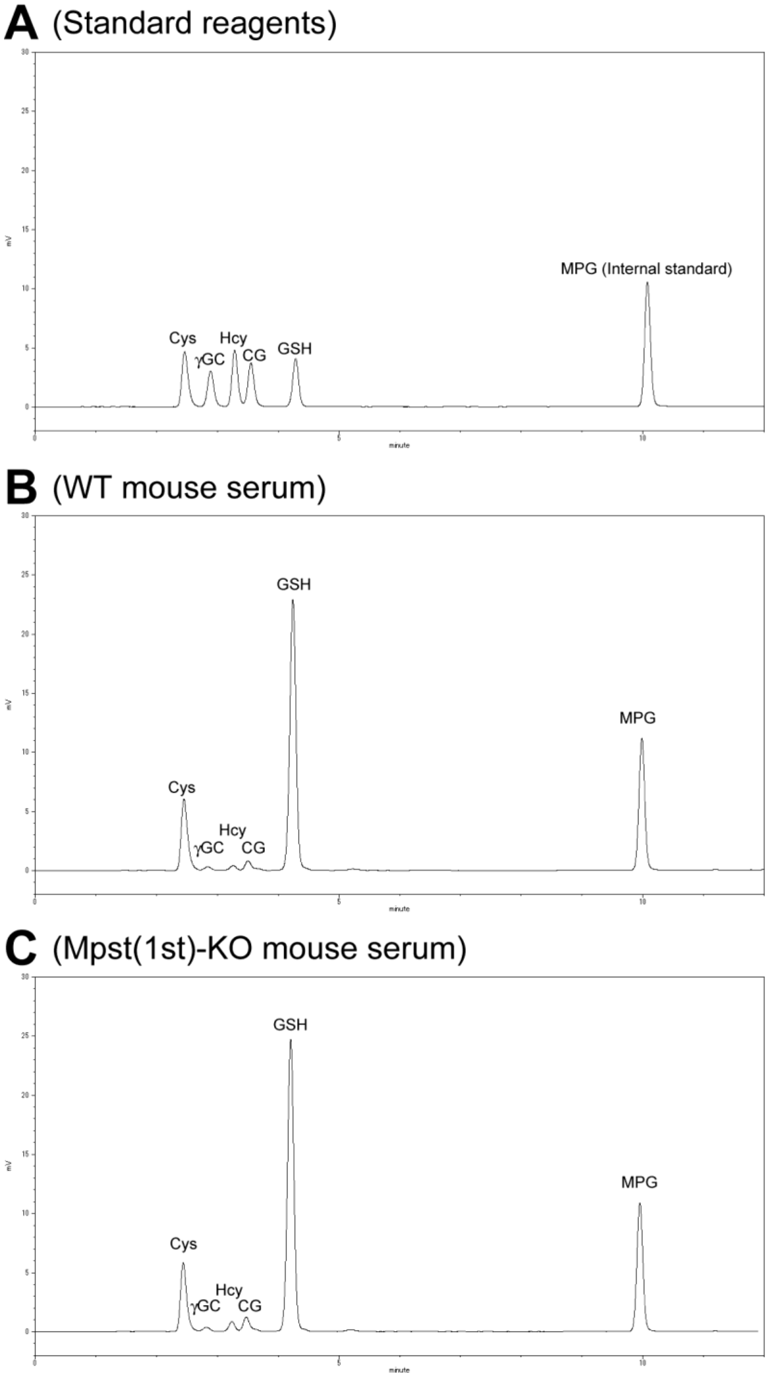

Supplementary Figure 5. Representative chromatograms in the measurements of thiol compounds in serum samples. Standard thiol compounds (A), WT mouse serum (B), and Mpst (1st)-KO mouse serum (C), were analyzed by SBD-F labeling and HPLC coupled with a fluorescence detector. γGC, (total) γ-Glu-Cys; CG, (total) Cys-Gly; Hcy, (total) homocysteine; GSH, (total) glutathione; MPG, *N*-(2-mercaptopropionyl)glycine (as the internal standard). The concentrations of the standard reagents in (A) are 200 μM for Cys, 100 μM for γGC, 40 μM for Hcy, 20 μM for CG and 40 μM for MPG.

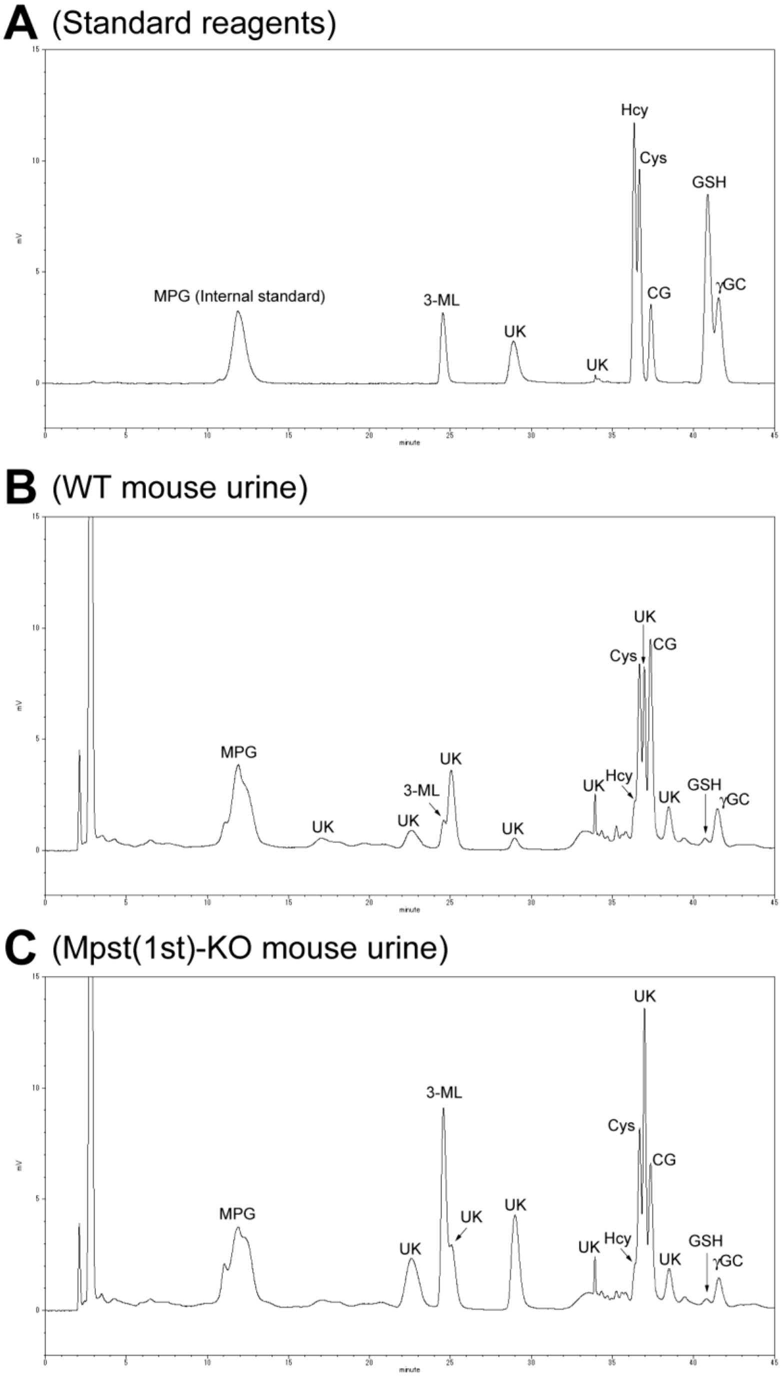

Supplementary Figure 6. Representative chromatograms for measurements of 3-mercaptolactate (3-ML) in serum. Standard thiol compounds (A), WT mouse urine (B), and Mpst (1st)-KO mouse urine (C), were analyzed by SBD-F labeling and HPLC coupled with a fluorescence detector. UK, unknown; 3-ML, 3-mercaptolactate; γGC, (total) γ-Glu-Cys; CG, (total) Cys-Gly; Hcy, (total) homocysteine; GSH, (total) glutathione; MPG, *N*-(2-mercaptopropionyl)glycine (as the internal standard). The concentrations of the standard reagents in (A) are 40 μM for MPG, 100 μM for 3-ML, 200 μM for Hcy, 1 mM for Cys, 40 μM for CG, 400 μM for GSH, and 200 μM for γGC.
